# Method for Detecting Pathology of Internal Organs Using Bioelectrography

**DOI:** 10.3390/diagnostics14100991

**Published:** 2024-05-09

**Authors:** Yulia Shichkina, Roza Fatkieva, Alexander Sychev, Anatoliy Kazak

**Affiliations:** 1 Department of Computer Science and Engineering, Saint Petersburg Electrotechnical University LETI, 197022 St. Petersburg, Russia; 2 Humanitarian Pedagogical Academy, V.I. Vernadsky Crimean Federal University, 295007 Simferopol, Russia

**Keywords:** bioelectrography method, detecting pathology of internal organs, machine-learning model

## Abstract

This article considers the possibility of using the bioelectrography method to identify the pathology of internal organs. It is shown that with the currently existing methods, there is no possibility of the automatic detection of diseases or abnormalities in the functioning of a particular organ, or of the definition of combined pathology. It has been revealed that the use of various classifiers makes it possible to expand the field of pathology and choose the most optimal method for determining a particular disease. Based on this, a method for detecting the pathology of internal organs is developed, as well as a software package that allows the detection of diseases of the internal organs based on the bioelectrography results. Machine-learning models such as logistic regression, decision tree, random forest, xgboost, KNN, SVM and HyperTab are used for this purpose. HyperTab, logistic regression and xgboost turn out to be the best among them for this task, achieving a performance according to the f1-score metric in the order of 60–70%. The use of the developed method will, in practice, allow us to switch to combining various machine-learning models for the identification of certain diseases, as well as for the identification of combined pathology, which will help solve the problem of detecting pathology during screening studies and lead to a reduction in the burden on the staff of medical institutions.

## 1. Introduction

The increase in the growth of computing power of modern computer systems accompanied by the decrease in their element base leads to the integration of knowledge, both in related subject areas and in the formation of multidisciplinary research, for example, in medical practice. However, this approach has a contradiction. On the one hand, this leads to an increase in the number of detected diseases. On the other hand, the processes of automating diagnosis require reducing the number of medical personnel involved in routing the patient. The requirements for highly specialized specialists who need to adapt to a rapidly changing hardware and software environment are also increasing. Against this background, there is a need to develop screening programs that allow you to quickly identify a particular pathology.

The purpose of the study described in this article was to increase the effectiveness of detecting the pathology of internal organs through data analysis using machine-learning methods. As a result, a method for detecting the pathology of internal organs using bioelectrography was developed. The method analyzes which datasets are needed to assess violations, identifies the patterns and correlations existing in them on the basis of which pathology is searched, and also identifies errors and data distortions that may affect the results of the processing. A distinctive feature of the developed method is the possibility of synthesizing new records, which allows you to solve the problem of data imbalance. This made it possible to develop a software package for the practical implementation of the above method and to form an ensemble of machine-learning models that identify pathology according to the description of GRV-grams.

The novelty of the proposed method consists of:

- the study of various machine-learning models for use in the detection of certain diseases using gas discharge imaging;
- the development of a method for detecting the pathology of internal organs using bioelectrography data, characterized by the use of machine-learning methods to identify the pathology of internal organs, which allows the use of a combination of different classifiers to determine combined pathology;
- the development of a model that allows you to obtain a dataset based on a sample of diseases, as well as generate training, validation and test kits in semi-automatic mode.

Using the developed method in practice will allow us to move to combining various machine-learning models to identify certain diseases, as well as to identify combined pathology, which will help solve the problem of detecting pathology during screening studies and reduce the workload of employees of medical institutions.

The remaining portions of this paper are organized as follows. Section 2 presents the results of the analysis of related work. Section 3 discusses the method of detecting the pathology of internal organs using bioelectrography, as well as the software package used in this work. Section 4 presents the experimental results from a dataset with a sample of 170 patients. Section 5 presents a discussion of the results obtained.

## 2. Related Work

Such methods include studies that allow, on the basis of the X-ray images and machine-learning methods used, us to identify the pathology of the respiratory organs [1,2,3]. The methods based on the classification of untreated lung sounds and the detection of pneumonia and chronic obstructive pulmonary diseases can be attributed [4,5,6]. A separate area is the work on the detection of oncological diseases [7,8,9]. The use of machine-learning methods also makes it possible to identify the pathology of the genitourinary system [1]. Image segmentation based on machine-learning models and neural networks makes it possible to identify and classify diseases of the gastrointestinal tract [10,11,12,13]. In terms of identification studies, possible heart diseases based on clinical data in different patients were considered in [14], but their application in practice relies on the complexity of the data and the presence of correlations between them. The listed methods have a high resource intensity in terms of the applied software and hardware. Summarizing the results [1,2,3,4,5,6,7,8,9,10,11,12,13,14], it is advisable to note that with almost all the methods, there is no possibility of automating the detection of pathology or deviations in the functioning of a particular organ (Table 1). In the table, a «+» sign indicates the presence of this property from the column header in the method from the first column in this source, «−» its absence.

Existing methods are usually limited to considering the pathology of only one of the body’s systems. As a rule, the methods for detecting combined pathology are not considered in studies [1,2,3,4,5,6,7,8,9,10,11,12,13,14,15,16,17,18,19,20,21,22,23,24].

One of the methods with low resource consumption and the ability to quickly identify diseases is the bioelectrography method based on gas discharge imaging (GDV) [15,16,17,18,19,20,21,22,23,24].

To identify relevant works, we, as well as the authors of [21], analyzed publications in the Google Scholar, ResearchGate and Elibrary databases using the following keywords: visualization of gas discharge (GDV), Kirlian effect, bioelectrography. The study revealed that during the period from 2000 to 2005, 9 works were published on the research topic; from 2005 to 2010, 48 works; from 2010 to 2015, 72 works; from 2015 to 2020, 51 works; and from 2020 to the present, 18 works. Thus, the peak of publications occurred in the period from 2010 to 2015 (72 works). From the works presented for analysis over the past decade, one can single out a study [17] aimed at establishing normative bioelectrography data for the healthy population of India in order to increase the accuracy of its measurement and interpretation, since according to the authors [17], the data for this population group differ from Europeans. In [18], causal relationships have been established between the parameters of gas discharge imaging and the main neuroendocrine adaptation factors, indicating the informativeness of this method. Using other methods, the authors of [19] reached a similar conclusion, in which it was shown that the method of gas-discharge imaging using a Bio-Well device makes it possible to determine the effect of diagnostic ultrasound on human homeostasis, which opens up prospects for using a Bio-Well device to assess the effects of various medical technologies, both diagnostic and therapeutic, on the human body.

In [20,21,22,23], a classification of the practical application of bioelectrography methods in medicine is provided: analysis of the vegetative status of the body and individual functional systems; monitoring of the body’s reactions to therapy; assessment of the likelihood of organ pathology. However, all the studies have a number of disadvantages, in particular:

- the difficulty in ensuring the completeness of the volume and quality of the sample of the studied data due to both obtaining the sample itself and decoding medical diagnoses and data;
- the lack of methods for detecting the correlation of organ dysfunction in combined pathology;
- the data transmitted from the bioelectrography device is stored in an unordered form and requires further processing;
- the marking of bioelectrography zones does not always correctly reflect possible pathologies of internal organs.

These problems require additional research to identify a particular pathology. The use of various classifiers allows you to increase the space of diseases and choose the most optimal method that identifies a particular pathology. To identify violations of internal organs in this work, a software package is proposed that allows you to obtain a dataset based on a sample of diseases, as well as to generate training, validation and test kits in automatic mode.

## 3. Materials and Methods

Before starting to process the data, it is necessary to conduct an analysis to determine which of the sets are necessary for the assessment of pathology, to derive patterns that allow you to determine the correlation of their characteristics, and to form an algorithm of actions to achieve the goal. This approach allows you to identify errors and data distortions that may affect the results of the processing.

To accomplish this, two types of data are generated, the type of data of the characteristics of the disease received from the patient and the type of data of the thresholds, the excess of which characterizes the presence of the disease. Next, to form a dataset, data are received from patients and their feature space is normalized with the removal of irrelevant features and outliers. This allows you to create 3 samples with data: training, validation and test. In the process of model learning, the set of applied models is refined. This makes it possible to leave only informative models from the formed complex, as well as to clarify the vectors of the data type responsible for detecting pathology and the vector identifying a particular pathology. Let us look at this method in more detail:

***Step 1***. The formation of a feature space of two types. The first type forms the characteristic space of bioelectrography characteristics, while the second type of data forms the characteristic space of pathology of internal organs. Comparing both types with each other in the following steps makes it possible to identify the characteristics of bioelectrography with a particular type of disease. Let us take a closer look at the formation of 2 types of datasets:

*Type 1:* data suggesting pathology of internal organs. For example, type 1 sets may represent data that can be easily obtained, but it is not possible to draw a conclusion based on them. The feature space of data characteristics of type 1 are denoted as:

(1) $$B_{s}=\left\{b_{1}{,\;b}_{2}{,\;\ldots b}_{i},\;\ldots {,\;b}_{s}\right\}$$

where $b_{i},\;i=\bar{1,s}$—are the characteristics of the feature space. For example, for bioelectrography, the element of the set $b_{1}$ may correspond to the number of pixels of the image of the GRV-gram of any zone of a certain finger, and $b_{2}$ to the radius of the inner circle of the GRV-gram zone, etc.

**Figure 1:**
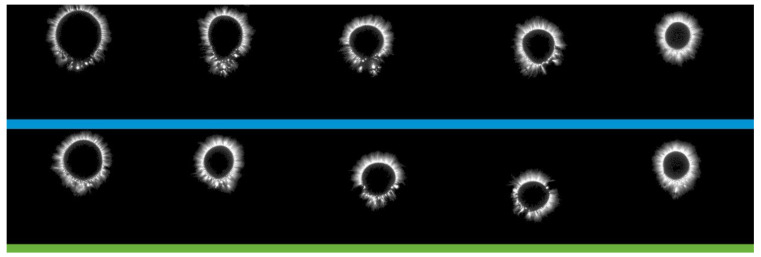
An example of a GRV-gram for a patient with kidney pathology.

For a more visual representation, it is advisable to provide an example of a snapshot of a gram (Figure 1).

The GRV-grams obtained were processed using computer vision algorithms. This made it possible to form the $B_{s}$ vector of the GRV-gram feature space. The vector contains about a thousand elements. The main ones include the following:

- Ellipse dimensions—the dimensions of the inscribed ellipse in X and Y in pixels;
- Radius of the inner circle—the radius of the inscribed circle in pixels;
- Area—the number of pixels of the sector image;
- Area (K)—the ratio of the glow area of the finger to the glow area of the calibration cylinder of the sector;
- Normalized area—the ratio of the area of the glow to the area of the inner oval of the sector;
- Intensity—the average intensity of all the pixels in the sector;
- Inner area—the total number of pixels in the inner oval of the sector;
- Internal noise—the number of noise pixels in the inner oval of the sector;
- Internal noise (%)—the ratio of the internal noise of the internal area of the sector as a percentage;
- Energy—the glow energy in joules (×10^−2^) of the sector;
- Energy (K)—the energy adjusted for the angular size of the sector;
- Shape coefficient (CF)—the ratio of the square of the length of the outer contour to the area of the image;
- Entropy coefficient (KE)—the ratio of the length of the outer contour to the length of the inner sector;
- Inner contour length—the length of the inner contour in pixels of the sector;
- Inner contour radius—the radius of the inner contour in pixels of the sector;
- Length of the outer contour—the length of the outer contour in pixels of the sector;
- Outer contour radius—the radius of the outer contour in pixels of the sector.

If the feature space is too large, it is possible to submit not all of them at once, but some of them can be used, based on algorithms for reducing the feature space.

*Type 2:* data of the feature space, which can be used to uniquely identify pathology, with the possibility of using them for machine-learning models. The second type of data may contain elements of many patterns of pathology description. Such patterns will be further designated as follows:

(2) $$C=\left\{c_{1}{,\;c}_{2}{,\;\ldots ,c}_{j},\;\ldots {,\;c}_{r}\right\}$$

where $c_{j},\;j=\bar{1,\;R}$—are the characteristics of the feature space identifying the pathology.

**Figure 2:**
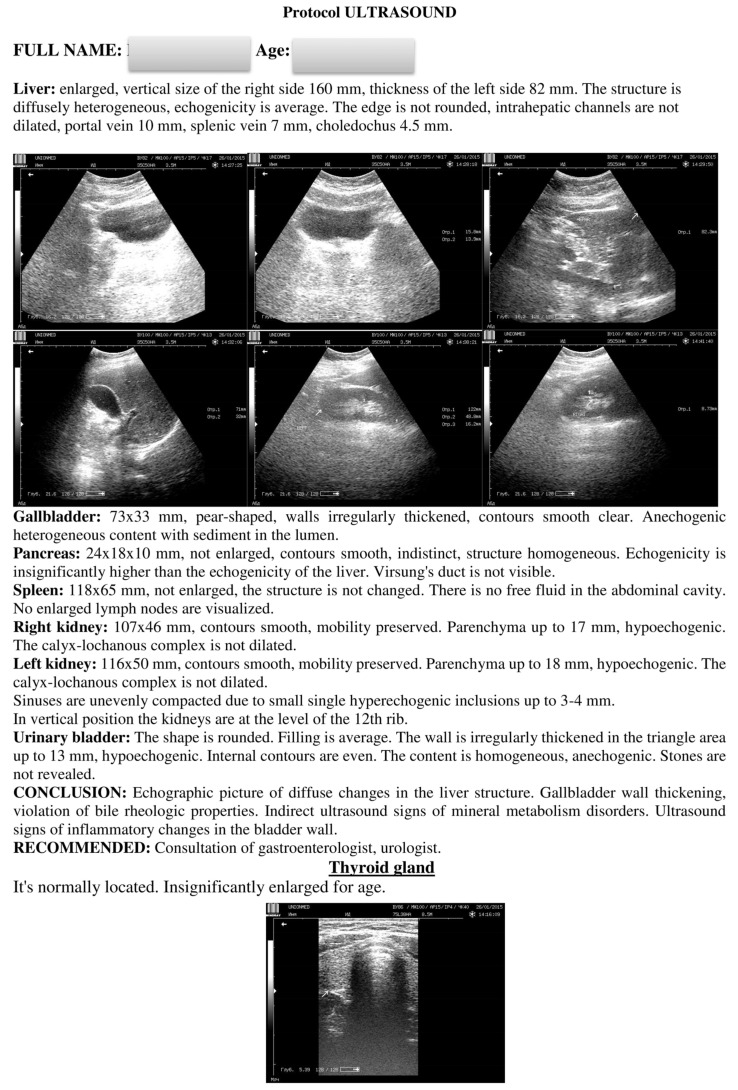

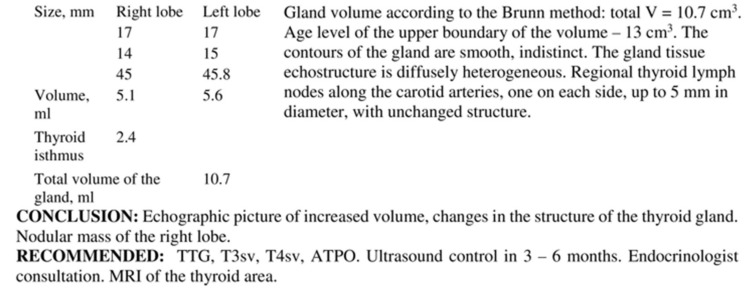
An example of an ultrasound protocol of the internal organs (liver, kidneys, spleen, adrenal glands, pancreas, etc., in Russian).

To perform this, the target signs of the pathology for each patient are taken from the conclusions of ultrasound diagnostics specialists (an example of the protocol is shown in Figure 2).

For example, the element $c_{1}$ may indicate that the patient has hepatomegaly, and $c_{2}$, diffuse changes in the structure of the thyroid gland.

In a particular case, it can be said that datasets from bioelectrography belong to type 1, and data from ultrasound belong to type 2. It should be clarified that both types should be collected based on data from the same patients, in quantity N.

***Step 2***. Identification of the patient’s characteristic space. This step is necessary to compare the individual characteristics of the patient obtained from bioelectrography with the target characteristics of his pathology. Then, for each patient, within the types selected in step 1, we leave only those that characterize his individual characteristics:

2.1. Type 1 data obtained from a special device is decomposed into elements of the feature space $B_{s}$ for each patient. That is, each patient will be characterized by their own characteristic space $B_{s}^{i}$, where $i=\bar{1,N}$ is the patient’s number. The order of the signs is the same in all the patients, but the values are different. For example, a type 1 data feature space for a patient i=1…N may look like this:

(3) $$B_{s}^{i}=\left\{b_{1}^{i}{,\;b}_{2}^{i}{,\;\ldots ,\;b}_{i}^{i},\;\ldots {,\;b}_{s}^{i}\right\}$$

2.2. For each patient, their own set of data feature spaces of type 2 is compiled:

(4) $$C^{i}=\left\{c_{2}^{i}{,\;c}_{5}^{i}{,\;c}_{8}^{i},\;\ldots {,\;c}_{k}^{i}\right\}$$

To clarify, the number i for the type 1 data and type 2 data should indicate the same patient. If the patient has no pathology, then the element of the set is skipped, for example, for a patient with $C^{i}=\left\{c_{2}{,\;c}_{5}{,\;c}_{8},\;\ldots {,\;c}_{k}\right\},\;k\;\leq \;r,$ signs of hepatomegaly pathology, the element is $c_{2}$ missing from the set.

***Step 3.*** Normalization of the data feature space for type 1. Since the data obtained for type 1 are heterogeneous, it becomes necessary to bring them to a single scale and range of values. To accomplish this, we introduce the operator $F_{\mathrm{norm}}$:

(5) $$F_{\mathrm{norm}}{:\;B}_{s}^{i}\;\to B_{\mathrm{sN}}^{i}$$

where $B_{\mathrm{sN}}^{i}$ is a set of normalized feature space for the *i* -th patient.

Normalization is necessary to convert the data into a single form, improve the convergence of the optimization algorithm and level the weights of features when training models in the future. The range of values during normalization and the normalization method itself depend on the task.

***Step 4.*** Reducing the dimension of the feature space to reduce the number of features used to describe objects, while preserving the most important properties of the data. To improve the training of models in the future and reduce the complexity of the data at this step, depending on the task, the dimension of the type 1 feature data space is reduced. Let us introduce the operator $F_{\mathrm{red}}$:

(6) $$F_{\mathrm{red}}{:\;B}_{\mathrm{sN}}\;\to B$$

Let us denote the elements $B^{i}$ for the *i*-th patient as follows:

(7) $$B^{i}=\left\{b_{1}^{i}{,\;b}_{2}^{i}{,\;b}_{3}^{i},\;\ldots {,\;b}_{n}^{i}\right\},\;i=\bar{1,\;N}$$

This step is necessary to simplify the data structure. Each gram is described by a large number of features, many of which are probably insignificant. Such preprocessing will simplify the process of learning classifiers.

After applying this operator, the number of elements in the set of attributes of type 1 data will decrease. Depending on the task, the operator $F_{\mathrm{red}}$ will be different. It is important to take into account that the operator $F_{\mathrm{red}}$ decreases the same signs for each patient. The operator can also change the basis of the feature space while reducing the feature space. Let us denote the size of the set B after applying the operator $F_{\mathrm{red}}$ with a symbol n.

***Step 5***. The introduction of a set $P^{i}$ describing the pathology of the patient number i. This step is necessary to specify and unify the structure of each sample element. The values for the presence or absence of pathology are set for each sample element. To achieve this, we denote the elements $P^{i}$ as follows:

(8) $$P^{i}=\left\{p_{1}^{i}{,\;p}_{2}^{i}{,\;p}_{3}^{i}{,\;\ldots ,\;p}_{z\;}^{i},\;\ldots {,\;p}_{m}^{i}\right\}$$

where $z=\bar{1,M.}$

For each element of the set $C^{i}$ obtained in step 2.1, we compare the set $P^{i}$ using the operator $F_{p}$. The order of pathologies should be the same $P^{i}$ for everyone:

(9) $$F_{p}:\;C\;\to P$$

Let us denote the size of the set P after applying the operator $F_{p}$ with the symbol m.

In a particular case, 1 is assigned to each element of the set $P^{i}$ when a pathology is detected, and 0 in its absence. For example, $P^{i}=\{0,\;1,\;0,\;1\}$, means: absence of heart pathology, presence of pathology of the left kidney, absence of liver pathology, presence of pathology of the spleen. Separately, we can note the case in which the value of the elements in the set can also be a symbol, meaning that there is no data on pathology (for example, if there are no liver data for a patient number k, then the third element $p_{3}^{k}$ of the set $P^{k}$ will be equal to this symbol. In the future, such a symbol will be designated as follows: “-”). The order of the elements in the set, their value and number, as well as the function itself, depend on the task and the selected machine-learning (ML) models.

***Step 6.*** Declaring a set of tuples to identify the pathology. To accomplish this, we form the matrix $M_{B}^{j}$, in which a row is responsible for each feature space of the set of characteristics obtained by bioelectrography in step 2.1, and the matrix $M_{P}^{j}$, in which the column values are assigned the presence or absence of pathology obtained in step 5. This allows you to proceed to the formation of datasets. Let us look at this step in more detail:

6.1. Defining a tuple ${(M}_{B}^{j}{,\;M}_{P}^{j})$ that consists of two elements:

(1) Matrices $M_{B}^{j}$ of dimension (N,n). The row i of the matrix represents the elements $p_{j}^{i}$, where $P^{i}$, i=1…N, j=1…m.

6.2. Formation of a set O consisting of tuples defined in step 6.1:

(10) $$O=\left\{\left(M_{B}^{1}{,\;M}_{P}^{1}\right),\;\left(M_{B}^{2}{,\;M}_{P}^{2}\right),\;\left(M_{B}^{3}{,\;M}_{P}^{3}\right),\;\ldots ,\left(M_{B}^{m}{,\;M}_{P}^{m}\right)\right\}$$

At the moment, all the matrices $M_{B}^{j}$ are the same. That is, the following is performed: $\forall j=1\ldots m,\;\forall k=1\ldots {m\;M}_{B}^{j}{=M}_{B}^{k}$. An example of matrices $M_{B}^{j}$ and $M_{P}^{j}$:

(11) $$M_{B}^{j}=\left(\begin{array}{ccc} b_{1}^{1} & \cdots & b_{n}^{1}\\ \vdots & \ddots & \vdots \\ b_{1}^{N} & \cdots & b_{n}^{N} \end{array}\right),\;\;\;M_{P}^{j}=\left(\begin{array}{c} p_{j}^{1}\\ \vdots \\ p_{j}^{N} \end{array}\right)$$

6.3. Formation of a set of operations on the matrices. For example, the operation $M\left[i\right]$ will mean taking the *i*-th row from the matrix $M_{B}^{j}\left[i\right]{=(b}_{1}^{i}{,\;b}_{2}^{i}{,\;b}_{3}^{i},\;\ldots {,\;b}_{n}^{i})$. The entry ${\mathrm{size}(M}_{B}^{j})$ means an action that will output the number of rows in the matrix $M_{B}^{j}$.

***Step 7.*** Delete irrelevant data and outliers. The initial data do not contain information about all the pathologies for each patient. For example, one group of patients has information about thyroid pathology, while the other does not. This step is necessary to remove irrelevant pathology data from patients who do not have information about the pathology in question. Each element $p_{j}^{i}$ where i=1…N, j=1…m can also have a symbol corresponding to the fact that there are no data (“-”). In this case, such data must be deleted. That is:

$$\forall i=1\ldots N,\;\forall j=1\ldots {m\;\mathrm{if}\;p}_{j}^{i}{=“-”\;\mathrm{then}\;\mathrm{delete}\;M}_{B}^{j}\left[i\right]{,\;M}_{P}^{j}\left[i\right]$$

It is also possible to delete explicit outliers or erroneous data.

***Step 8.*** Refinement of the characteristic data space of type 1, taking into account data of type 2.

The matrix $M_{P}^{j}$ corresponds to a set of pathologies of the internal organs. At this step, if necessary, you need to reduce the character space of the lines $M_{B}^{j}$ but take into account $M_{P}^{j}$. Let us introduce a set of operators: $F_{\mathrm{rep}}$

(12) $$F_{\mathrm{rep}}=\left\{F_{\mathrm{rep}}^{1}{,\;F}_{\mathrm{rep}}^{2}{,\;F}_{\mathrm{rep}}^{3},\;\ldots {,\;F}_{\mathrm{rep}}^{m}\right\}$$

Each operator is responsible for its own tuple of the set *O*:

(13) $$F_{\mathrm{rep}}^{j}:\;\left(M_{B}^{j}\left[i\right]{,\;M}_{P}^{j}\left[i\right]\right)\to M_{B}^{j}\left[i\right],\;i=1\ldots N,\;j=1\ldots m$$

After applying the operator $F_{\mathrm{rep}}^{j}$, the number of elements in the row $M_{B}^{j}\left[i\right]$ will decrease. It should be noted $F_{\mathrm{rep}}^{j}$ that it reduces the same elements for everyone i=1…N.

For example, $M_{P}^{3}$ may be “responsible” for liver pathology. Based on this, it is necessary to reduce the feature space of the matrix rows $M_{B}^{3}$, taking into account the fact that these data will reveal liver pathology.

***Step 9***. Forming data samples. At this step, for further training of the ML models, it is necessary to form 3 samples with data: training, validation and test. To accomplish this, for each $M_{P}^{j}$ and $M_{P}^{j}$, j=1…m, three disjoint lists of numbers from 1 to ${\mathrm{size}(M}_{P}^{j})$ are formed in such a way that the number of elements in the list when they are combined is equal ${\mathrm{size}(M}_{P}^{j})$. These 3 sets will be denoted by:

– set –$Z_{\mathrm{train}}^{j}$—set indexes of the training sample, the number of elements is approximately 50–80% of ${\mathrm{size}(M}_{P}^{j})$;
– set $Z_{\mathrm{val}}^{j}$—set indexes of the validation sample, the number of elements is approximately 10–25% of ${\mathrm{size}(M}_{P}^{j})$;
– set $Z_{\mathrm{test}}^{j}$—set indexes of the test sample, the number of elements is approximately 10–25% of ${\mathrm{size}(M}_{P}^{j})$.

The number of elements in the set varies depending on the number N.

***Step 10***. Creating a set of synthetic data. If there are little data (equivalent to the fact that $Z_{\mathrm{train}}^{j}$ indicates there are few elements), then the process of synthesizing new records and augmentation is carried out. It is important to note that only the matrix strings ${\{M}_{B}^{j}\left[i\right]\;|\;i\;\in \;Z_{\mathrm{train}}^{j}\}$ can be synthesized and augmented. The choice of synthesis and augmentation method depends on the field of work, data, and ML models.

To achieve this, we introduce an operator $F_{\mathrm{syn}}$ that will form a new tuple along the rows of the matrix:

(14) $$F_{\mathrm{syn}}{:\;\{(M}_{B}^{j}\left[i\right]{,\;M}_{P}^{j}\left[i\right])\;|\;i\;\in {\;Z}_{\mathrm{train}}^{j}\}\;\to {(M}_{\mathrm{Bsyn}}^{j}{,\;M}_{\mathrm{Psyn}}^{j})$$

We assign a new index equal to N+1 to such a tuple and add it to the set $Z_{\mathrm{train}}^{j}$, and then we increment it N. Then, we add a row $M_{\mathrm{Bsyn}}^{j}$ to the matrix $M_{B}^{j}$, and a row $M_{\mathrm{Psyn}}^{j}$ to the matrix $M_{P}^{j}$.

The synthesis of new data can also help to cope with the problem of imbalance. There are two ways to solve the problem here: to make the major and minor classes equal, or to make the minor class major.

***Step 11***. Training of ML models based on the data obtained in steps 9–10. At this step, a general list of ML models with different parameters is compiled. Each of these models will be further trained and validated. At this step, you need to create many models of ML:

(15) $$\mathrm{ML}=\left\{v_{1}{,v}_{2}{,v}_{3},\ldots {,v}_{L}\;\right\}$$

where $v_{L}$- is the machine-learning model. The choice of the model depends on the subject area and the data. At this stage, you also need to decide on the type of metrics (precision, recall, f1-score or another). After composing the set ML, it is advisable for each element of the set O (10) to train each model from (15) on ${\{(M}_{B}^{j}\left[i\right]{,\;M}_{P}^{j}\left[i\right])\;|\;i\;\in {\;Z}_{\mathrm{train}}^{j}\}$, selecting hyperparameters on ${\{(M}_{B}^{j}\left[i\right]{,\;M}_{P}^{j}\left[i\right])\;|\;i\;\in {\;Z}_{\mathrm{test}}^{j}\}$, with accuracy checking on ${\{(M}_{B}^{j}\left[i\right]{,\;M}_{P}^{j}\left[i\right])\;|\;i\;\in {\;Z}_{\mathrm{test}}^{j}\}$. Based on these results, it is advisable to choose the best model for each element of the set O based on the selected metric.

***Step 12.*** Refinement of the set of applied models. Here, from the entire set of models obtained in the previous step, those that are the most informative for each type of pathology are selected. This allows you to form an ensemble of models with the pathology necessary for analysis. After choosing the best models, they must be combined into an ensemble V:

$$V=\left\{v_{1}{,v}_{2}{,v}_{3},\ldots {,v}_{m}\right\}$$

where each ML, $v_{i}$ model, j =1…m is responsible for a certain pathology.

Now, when receiving new data of type 1, they can be transferred to the ensemble V, which in turn forms a vector P:

(16) $$P=\left\{p_{1}^{\mathrm{pred}}{,p}_{2}^{\mathrm{pred}}{,p}_{3}^{\mathrm{pred}},\ldots {,p}_{m}^{\mathrm{pred}}\right\}$$

where each element $p_{j}^{\mathrm{pred}}$, j=1…m, of the vector P describes the presence of pathology. For example, if the vector $p_{3}^{\mathrm{pred}}$ is 1, then this may mean, for example, liver pathology.

## 4. Software Package for Detecting Pathology of Internal Organs

**Figure 3:**
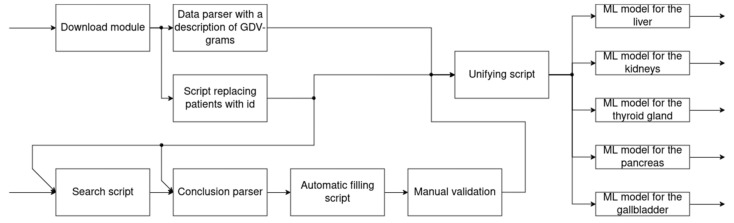
Software for detecting the pathology of internal organs.

To identify the pathology of the internal organs, a software package was formed (Figure 3), which includes a module with the implementation of gradient boosting: “xgboost”; libraries for ML “scikit-learn”; “pandas” and “NumPy”; “imblearn”; and “pyplot”. The following models were chosen to compose the ensemble: decision tree; KNN; logistic regression; random forest.

***Logistic regression*** is a machine-learning method used to predict the probability of an observed object belonging to a certain class. The goal of logistic regression is to find the optimal hyperplane that separates objects of two classes as accurately as possible based on the values of their features. This hyperplane can be used to predict the probability of an object belonging to one of the classes.

To train the logistic regression model, the maximum likelihood method is used, which allows you to find such values of the model parameters at which the probability of the observed data will be the maximum.

An important advantage of logistic regression is the ability to interpret the results. The coefficients of the model, which determine the contribution of each feature to the prediction of a class, can be used to determine which features are most important for classifying objects.

A ***decision tree*** is a machine-learning method that is a tree in which each node represents a decision when choosing a specific feature to divide data into smaller groups.

The process of building a tree begins with the root node, which contains all the available data. The tree is then divided into two or more branches based on the value of the selected attribute to divide the data into smaller groups. Each of these new nodes can also be divided into smaller groups using a different attribute, and so on until the specified stopping criteria are reached. The result of the tree is rules describing how the data should be divided into groups in order to obtain the best prediction result for the target variable.

The decision tree has the property of a simple interpretation of the received rules.

***Random forest*** is a machine-learning algorithm used for tasks such as classification or regression. It is an ensemble of decision trees, that is, it consists of several trees, each of which solves a classification or regression problem for the input data.

When classifying data, a random forest uses majority voting—each tree “votes” for a certain class, and the final result is chosen by majority vote.

***Gradient boosting*** is a machine-learning method used to solve classification and regression problems. It is based on the idea of combining several weak learning models, such as decision trees, to create a stronger model.

Unlike a random forest, where each tree is created independently of the others, in gradient boosting, each new tree is created by taking into account the errors of previous trees. The gradient-boosting algorithm builds a new decision tree at each iteration, which takes into account the errors of the previous model.

One of the most popular and effective implementations of the gradient-boosting algorithm is XGBoost.

The ***support vector machine*** (SVM) method is a machine-learning method that is used for both classification and regression tasks. It is based on the construction of an optimal hyperplane that maximally separates the data of two classes in a multidimensional feature space. That is, the separating hyperplane is located at the maximum distance from the classes in the feature space.

In addition, a SVM can work with non-linearly separable data by using cores that project data into a higher-dimensional space.

***HyperTab*** is a classifier for small datasets based on a hypernet. The smaller the dataset, the greater the advantage of HyperTab over other algorithms. The principle of operation of HyperTab is the generation of target networks by a hypernetwork, thus obtaining an ensemble of networks. The learning parameters are the hypernet parameters. To form an element of the ensemble, an augmentation mechanism is used for a subset of points in the feature space, which allows you to actually expand the original dataset. Augmentation in HyperTab terminology refers to the imposition of a mask on the features of an element.

The individual software modules included in the complex allow the following:

- Download module—download from the program where the GRV-grams and their description for each patient are stored;
- A data parser with a description of the GRV-grams—convert data describing the GRV-grams into a feature space (2);
- A script replacing the full name of patients with an ID—assign a unique ID to each patient and organize a repository with records about it according to (3).
- A search script and a conclusion parser—search throughout the array of documents with the conclusions of ultrasound diagnostics specialists are those that are necessary for training ML models according to (4);
- Automatic filling script—add columns of pathologies for each organ to the table with patient IDs and their conclusions. These columns have a Boolean value: “yes” or “no”. “Yes” means that there is a pathology (8).
- The unifying script is to combine tables, forming all the data into one view, in which the columns contain: patient IDs, descriptions of their GRV-grams and pathologies (11). Machine-learning models can work with such a table in the future.

Figure 3 shows the result of the obtained vector (16), formed at step 12, defining the entire set of pathologies, where each pathology has its own place in the vector. Furthermore, the characteristic responsible for a certain pathology is extracted from this vector, and a decision is made on the presence or absence of it.

The chosen scripts and modules of the software package provide automation and standardization of the data processing and analysis process, which simplifies the work of specialists and increases the efficiency of their activities. It also allows you to significantly reduce the time spent on data preparation and analysis, which is especially important in conditions of intensive medical practice.

## 5. Experimental Results and Analysis

**Figure 4:**
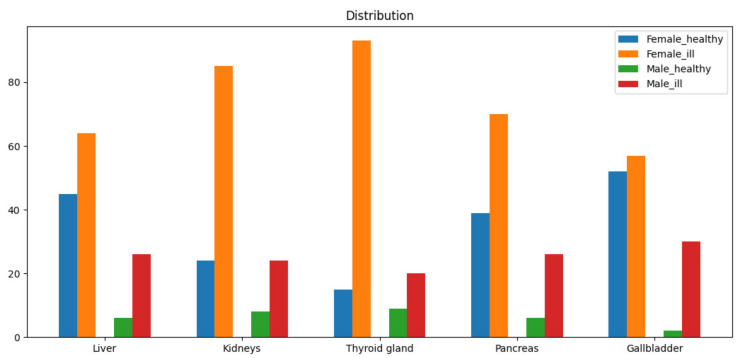
Histogram of the distribution of the pathologies of the internal organs.

The table obtained at the last step of the combining script (Figure 3) allows you to proceed to the selection of machine-learning models. To train machine-learning models, a dataset was compiled based on a sample of 170 patients’ diseases. The data were provided by the medical center, and the sample included 130 women and 40 men. The distribution of the pathologies of the internal organs is shown in Figure 4.

Several conclusions can be drawn from the histogram. The data are highly unbalanced. There are more patients with pathology than healthy ones, and significantly so for the following organs: liver, kidneys, thyroid gland, pancreas and gallbladder (Table 2 shows a numerical description of the distribution of patients). Such an imbalance must be taken into account when training ensemble models. The data describing the spleen is critically unbalanced. Pathology is not observed in a large majority of patients (which is true). With such a strong imbalance, it is impossible to train the model, and therefore, the spleen data will not be taken into account in the future.

Since each object in the feature space is characterized by over 1000 features, it is obvious that there is a need to reduce the feature space. In general, data modification can be divided into three directions: leave the dataset unchanged; reduce the feature space; synthesize new data. We would like to note right away that with regard to the validation and test datasets, there can be no question of synthesizing new objects in the feature space. Therefore, the third direction was applied exclusively to the training dataset. The dataset was processed using StandardScaler from scikit-learn 1.4.2 (https://scikit-learn.org/stable/modules/generated/sklearn.preprocessing.StandardScaler.html, accessed on 10 February 2024). To reduce the feature space, the methods of factor analysis and principal component analysis were used. When synthesizing new data, the following strategies were used: alignment of the major and minor classes; change of the minor class to the major one. The second strategy is used in some cases, as presented in [20]. Weights were also used to combat the unbalance when training models and selecting metrics. The distribution of data in the training, validation and test datasets is shown in Table 3.

From the table, you can see that the imbalance of classes in the datasets for the gallbladder and liver is minimal compared to the rest. This means that it will be easier for machine-learning models to learn from them. It should be noted that with the thyroid, the situation is the opposite—there is an imbalance of classes in the dataset, which is why the machine-learning model can very quickly choose a strategy to always predict the pathology class.

The resulting dataset made it possible to train machine-learning models to identify the pathology of internal organs, particularly the pathology of the gastrointestinal tract, kidneys and thyroid gland and spleen. The following classifiers of machine-learning methods were selected for the study: decision tree; KNN; logistic regression; random forest; SVC; XGBoost; HyperTab. The learning outcomes are presented in Table 4.

At the training stage, the dataset was split randomly. Also, during the splitting, preprocessing was performed, which included alignment, normalization, and reduction of the feature space. This made it possible to create several different datasets. Cross-validation performed with the parameter k = 5 was used, but it showed approximately the same results as its absence. This made it possible to decide to leave the division into three subsamples, in which the training and validation were changed, and the test was fixed. The training was conducted in several rounds. It was not possible to identify a universal model for all types of pathology.

Different models predict good results for each individual pathology. In the results were selected models that identified each pathology, the data on which are summarized in Table 4.

From Table 3, we can conclude that the presented models have good values for both precision and recall. The best results are shown by models where the class imbalance is the smallest. In the thyroid gland, where the class imbalance is high, the recall metric is unsatisfactory.

The KNN classifier and SVM were used, but they were excluded from further analysis because they did not show the best results in detecting pathology. Together with the fact that the training of these models is performed over a sufficiently long time interval, it did not make sense to use them in the future. The GDA and Naïve classifiers were also excluded from consideration under the assumption that the features that describe the objects of the dataset are related.

**Figure 5:**
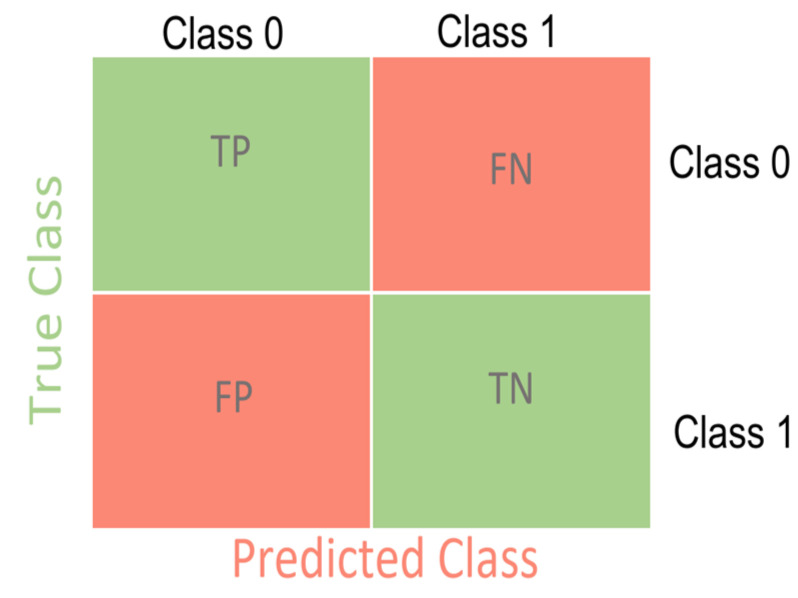
Confusion matrix.

The effectiveness of the training was assessed using the constructed histograms of the distribution of diseases and metrics: precision, recall, f1-score. In the process of predicting the model, an error matrix (confusion matrix) was compiled, an example of which is shown in Figure 5, where TP is the number of correctly predicted elements of class 0; TN is the number of correctly predicted elements of class 1; and FP is the number of elements that the model assigned to class 0, but this element actually belongs to Class 1. FN is the number of elements that the model assigned to Class 1, but this element actually belongs to class 0.

The error matrix expands if there are more than two classes. The effectiveness of the training can be assessed using metrics: precision, recall, f1-score. The metrics were calculated using the following formulas:

$$\mathrm{precision}=\frac{\mathrm{TP}}{\mathrm{TP}+\mathrm{FP}},$$

$$\mathrm{recall}=\frac{\mathrm{TP}}{\mathrm{TP}+\mathrm{FN}},$$

$$f1=\frac{2\;\cdot \;\mathrm{precision}\;\cdot \;\mathrm{recall}}{\mathrm{precision}+\mathrm{recall}}$$

The effectiveness of the training was assessed using the constructed histograms of the distribution of diseases and metrics: precision, recall, f1-score. The average value of the metrics (the third row of values in Table 4) was considered as an arithmetic mean and weighted as half of the sum of the metrics for individual classes multiplied by the coefficients depending on the unbalance of classes.

## 6. Discussion

Analyzing the data obtained, we can say that the percentage of errors is lower in those models where the distribution of the number of patients with pathology and healthy patients approaches normal.

The use of the HyperTab classifier has shown the possibility of its application for small tabular datasets based on a hypernet. To form an element of the HyperTab ensemble, the augmentation mechanism of a subset of the feature space points was used. Augmentation in HyperTab terminology refers to the imposition of a mask on the attributes of an element. This model has shown good results, which suggests the possibility of using neural networks where the input data will be a GRV-gram image.

However, it must be borne in mind that learning and using machine learning and neural network methods require large computational resources, and they also have weak interpretability. In this regard, this method can only be used if there are enough datasets: both the GRV-gram and the target attribute for each object in the sample. This requirement applies to every pathology, since data preprocessing and machine-learning models are used differently from case to case. For example, the analysis of the data in Table 2 showed the possibility of using this method with restrictions on the ratio of the number of patients with pathology of the internal organs and patients without such pathology. It can be seen from the table that there is an imbalance in the data that is due to the fact that there are too many patients with pathologies in the sample in relation to healthy ones. Models that were trained on those datasets where there is an “imbalance” (kidneys and thyroid gland) poorly identify a class of patients who do not have this pathology. Despite this, the application of the method has shown the possibility of its use and extension to other classes of tasks related to image processing.

The limitations of the method include non-specific limitations that are characteristic of all machine-learning methods, such as the following:

1. Necessity of large computational resources, as machine-learning methods require a significant amount of computational resources for training and data processing;
2. Poor interpretability of results when a new pathology arises that has not been previously described or is not available in the dataset.
3. The method also has specific limitations:
4. The requirement of a sufficient number of datasets of both GRV-grams and the target trait for each subject in the sample. This requirement applies for each pathology because different data preprocessing and machine-learning models are used for different types of ensembles.
5. The requirement for the balance of patients with pathologies and healthy patients in the dataset. Models that have been trained on those datasets where there is an “imbalance” are poor at identifying the class of patients who do not have the pathology.

The use of the developed method will, in practice, allow us to switch to combining various machine-learning models for the identification of certain diseases, as well as the for identification of combined pathology.

Further areas of research should include the formation of multiple zones of patients’ GRV-grams, with the solution of the problem of their clustering for the possibility of determining a particular pathology or their combinations based on machine-learning models. This will allow us to move on to solving the problem of detecting pathology during screening studies, which will lead to an increase in the detection of diseases and a reduction in the burden on the staff of medical institutions.

## Figures and Tables

**Table 1:** Comparison of methods for determining human organ pathologies.

Method	The Possibility of Detecting a Combined Pathology	Assessment of Correlation with Other Pathologies	Resource Intensity of the Method	Automation of Pathology Detection
Machine-learning methods for detecting respiratory pathology	+ [1]/− [2,3]	+ [1]/− [2,3]	+ [1–3]	− [1–3]
Methods based on the classification of pulmonary noises	− [4–6]	− [4–6]	+ [4–6]	− [4–6]
Detection of oncological diseases	+ [8,9]/− [7]	+ [9]/− [7,8]	+ [7–9]	− [7–9]
Machine-learning methods for detecting pathology of the genitourinary system	+ [1]	+ [1]	+ [1]	− [1]
Diseases of the gastrointestinal tract	− [10–13]	+ [11,12]/− [10,13]	+ [10–13]	− [10–13]
Detection of cardiovascular diseases	− [15]	+ [15]	+ [15]	+ [15]

**Table 2:** Distribution of diseases (imbalance).

	Liver	Kidneys	Thyroid Gland	Pancreas	The Gallbladder
Healthy	51	32	24	45	54
Pathology	90	109	113	96	87
Total relevant data	141	141	137	141	141
There is no data	29	29	33	29	29

**Table 3:** Distribution of survey results by dataset.

Dataset	Pathology	Liver	Kidneys	Thyroid Gland	Pancreas	The Gallbladder
Training (60%)	Healthy	31	19	14	27	32
	Pathology	54	65	68	58	52
Validation (15%)	Healthy	8	5	4	7	8
	Pathology	14	16	17	14	13
Test (25%)	Healthy	12	8	6	11	14
	Pathology	22	28	28	24	22

**Table 4:** Learning outcomes of an ensemble of machine-learning models.

Model	Metrics					
	Organ	Precision	Recall	F1-Score	Type of Feature	Confusion Matrix
HyperTab	Liver	0.53	0.67	0.59	Pathology is not revealed	[[ 8 4] [ 7 15]]
		0.79	0.68	0.73	Pathology revealed	
		0.66	0.67	0.66	Average	
		0.70	0.68	0.68	Weighted	
Logistic Regression	Kidneys	0.67	0.50	0.57	Pathology is not revealed	[[ 4 4] [ 2 26]]
		0.87	0.93	0.90	Pathology revealed	
		0.77	0.71	0.73	Average	
		0.82	0.83	0.82	Weighted	
HyperTab	Thyroid gland	0.75	0.50	0.60	Pathology is not revealed	[[ 3 3] [ 1 27]]
		0.90	0.96	0.93	Pathology revealed	
		0.82	0.73	0.77	Average	
		0.87	0.88	0.87	Weighted	
XGB Classifier	Gallbladder	0.64	0.64	0.64	Pathology is not revealed	[[ 9 5] [ 5 17]]
		0.77	0.77	0.77	Pathology revealed	
		0.71	0.71	0.71	Average	
		0.72	0.72	0.72	Weighted	
Decision Tree	Pancreas	0.47	0.82	0.60	Pathology is not revealed	[[ 9 2] [10 14]]
		0.88	0.58	0.70	Pathology revealed	
		0.67	0.70	0.65	Average	
		0.75	0.66	0.67	Weighted	

## Data Availability

There are no associated data to be shared related to this work.
